# Nanomaterials for Molecular Detection and Analysis of Extracellular Vesicles

**DOI:** 10.3390/nano13030524

**Published:** 2023-01-28

**Authors:** Mitchell Lee Taylor, Anthony Gregory Giacalone, Kristopher Daniel Amrhein, Raymond Edward Wilson, Yongmei Wang, Xiaohua Huang

**Affiliations:** Department of Chemistry, The University of Memphis, Memphis, TN 38152, USA

**Keywords:** extracellular vesicle, exosome, nanomaterials, molecular detection, cancer, diagnostics

## Abstract

Extracellular vesicles (EVs) have emerged as a novel resource of biomarkers for cancer and certain other diseases. Probing EVs in body fluids has become of major interest in the past decade in the development of a new-generation liquid biopsy for cancer diagnosis and monitoring. However, sensitive and specific molecular detection and analysis are challenging, due to the small size of EVs, low amount of antigens on individual EVs, and the complex biofluid matrix. Nanomaterials have been widely used in the technological development of protein and nucleic acid-based EV detection and analysis, owing to the unique structure and functional properties of materials at the nanometer scale. In this review, we summarize various nanomaterial-based analytical technologies for molecular EV detection and analysis. We discuss these technologies based on the major types of nanomaterials, including plasmonic, fluorescent, magnetic, organic, carbon-based, and certain other nanostructures. For each type of nanomaterial, functional properties are briefly described, followed by the applications of the nanomaterials for EV biomarker detection, profiling, and analysis in terms of detection mechanisms.

## 1. Introduction

Tissue biopsy is the gold standard method in the clinic for cancer diagnostics. It plays an important role in tailoring best treatment options for individual patients. However, tissue biopsy is invasive, costly, impractical for repeated testing, and unattainable for some cancer types. In addition, tumors are heterogeneous and evolve over time. Thus, biopsy data collected from limited tumor regions can be biased and can mislead clinical decisions. Additionally, the primary tumor is not accessible after surgery, inhibiting treatment monitoring. The limitations in tissue biopsy have driven the development of liquid biopsy, a means to detect and analyze cancer biomarkers in body fluids such as blood and urine.

Current liquid biopsy uses protein, nucleic acid, or tumor cells as the biofluid analytes [1]. Extracellular vesicles (EVs), including exosomes and microvesicles, have emerged as novel analytes for liquid biopsy for diagnostics, prognosis, and treatment monitoring of cancer and other diseases, due to their unique attributes [2,3,4,5]. EVs are membrane-bound vesicles that are continuously released into the extracellular environment by virtually all cells, with exosomes of endosomal origins and microvesicles from the plasma membrane [5,6,7]. They carry DNA, mRNA, and various proteins, such as tetraspanins, receptors, and adhesion molecules, which are reflective of their parental cells [5,8,9,10]. EVs from tumor cells can transfer oncogenic factors through intercellular communication and regulate angiogenesis, immunity, and metastasis, to promote tumorigenesis and progression [11,12,13,14,15,16,17]. Tumor-derived EVs enter the blood and many other body fluids, such as urine, saliva, and ascites, and cerebrospinal fluid [18,19,20,21,22]. Thus, EVs offer a robust new source of biomarkers for noninvasive liquid biology in medicine [23].

EVs are typically collected and isolated from body fluids using density- (differential centrifugation and precipitation), size-, and affinity-based methods, as well as microfluidics, which uses one or more of the aforementioned principles [24]. These methods eliminate the proteins and cell-debris or capture one specific molecular EV subtype for downstream analysis. They also concentrate EVs to facilitate subsequent analysis. 

While size and morphology can be readily characterized using dynamic light scattering (DLS), nanoparticle tracking analysis (NTA), and electron microscopes, the molecular detection and analysis of EVs, however, are challenging, due to their small sizes and correspondingly low number of analytes per EV (down to a single digit for small exosomes). In the past decade, a variety of characterization methods have been used, including traditional protein and nucleic acid methods such as polymerase chain reaction (PCR), enzyme-linked immunosorbent assay (ELISA), Western blot, and flow cytometry, as well as emerging methods that involve advanced methodologies, materials, and/or devices [25]. Compared to traditional methods, these advanced methods improve EV’s molecular detection and analysis, in terms of sensitivity, simplicity, and efficiency, and thus are promising for use in clinical settings.

**Figure 1:**
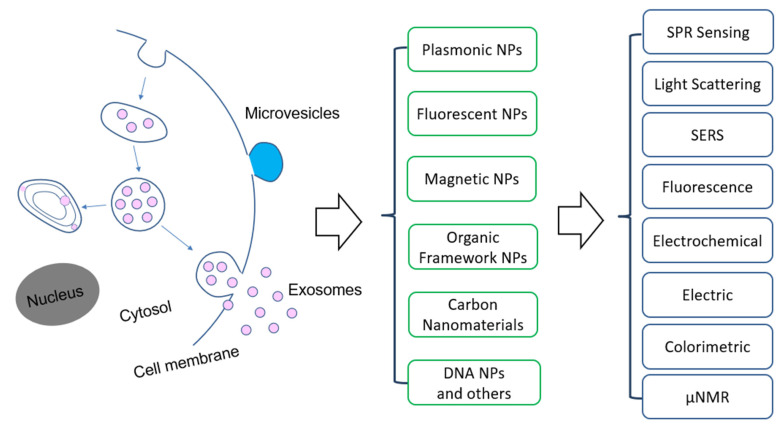
Overview of the application of nanomaterials and detection methods for extracellular vesicles. Abbreviation: NPs—nanoparticles, SPR—surface plasmon resonance, SERS—surface enhanced Raman scattering, µNMR: micro-nuclear magnetic resonance.

Among the emerging molecular characterization techniques, nanotechnology is playing an important role, due to the unique structure and functional properties of materials at the nanometer scale [26,27,28,29,30,31]. Nanomaterials exhibit exceptional functional properties that are often not available from discrete molecules and bulk materials. Nanostructures have a large surface-to-volume ratio that allows for highly efficient target interactions. The structure and functional properties can be used to develop new assays to overcome the limitations of the traditional ones. For molecular EV detection, a variety of nanomaterials have been used (Figure 1). For example, Lee and colleagues developed a nano-plasmonic exosome (nPLEX) assay based on surface plasmon resonance (SPR) sensing, with periodic nanohole to detect surface protein markers on exosomes and with a sensitivity 10^4^ fold higher than Western blot and 10^2^ fold higher than ELISA [32]. Hu and co-workers developed a different nanoplasmonic approach that can quantify tumor-derived exosomes in plasma with microliter-scale samples using the SPR light scattering properties of gold (Au) nanoparticles (NPs) in spherical and rod shape [33]. Huang’s group reported a surface-enhanced Raman scattering (SERS) method with gold nanorods (AuNRs) that achieved a limit of detection (LOD) of 2 × 10^6^ exosomes per milliliter [34].

This review will summarize and discuss the nanotechnology-based methods and devices that have been developed within past decade for molecular EV detection and analysis, aiming at cancer research and applications. Differently from previous reviews, we categorize the methods based on the type of nanostructures used, including plasmonic, fluorescent, magnetic, organic, and carbon-based nanomaterials. For each type of nanomaterials, functional properties are briefly described, followed by the applications for EV’s biomarker detection, profiling, and analysis in terms of the detection mechanisms.

## 2. Plasmonic Nanostructures

When conduction electrons at the interface of two materials, where the real part of the dielectric function changes sign across the interface, are stimulated by incident light, a resonant oscillation of those electrons, known as SPR, occurs. The generated surface plasmon polariton, which is a non-radiative electromagnetic wave that propagates on the dielectric interface, is sensitive to small changes at this boundary, such as the absorption or disassociation of molecules or small biological entities such as proteins [35]. This characteristic has made SPR an attractive technology for researchers interested in molecular and biological sensing, including the detection of exosomes and microvesicles. 

When the dimensions of the plasmonic materials decrease to the nanoscale, the SPR is confined to the surface of the nanomaterials. This localized SPR (LSPR), which is much stronger than SPR, can be obtained either with patterned nanostructures on a two-dimensional substrate or colloidal NPs of novel metals, such as Au and silver (Ag). In particular, for decades, colloidal AuNPs have been attractive nanomaterials across many fields, from materials science to biomedical applications, owing to their intriguing LSPR-enhanced optical properties [36,37,38,39,40,41]. This LSPR is size, shape, and structure sensitive, which leads to tunable optical properties, from the visible to the near infrared (NIR) region [42,43,44,45,46,47]. For example, when the shape of AuNPs is changed from a solid sphere to a rod, the LSPR is red shifted to the NIR region, a tissue penetrative window suitable for in vivo photothermal cancer therapy [48,49,50]. Due to the LSPR, AuNPs absorb and scatter light orders of magnitude stronger than non-metallic NPs and individual organic dyes [51]. Under dark field imaging, AuNPs appear as bright fluorescence analogs, due to their strong light scattering property [52,53,54]. Unlike fluorophores, AuNPs do not photo-bleach. Thus, AuNPs have been used as excellent contrast agents for bioimaging. The LSPR induces a strong electromagnetic field around the NPs, which can enhance the Raman scattering signals of adsorbed molecules [55]. SERS spectroscopy offers a fingerprint tool for detection and quantification of targeted molecules of interest [56]. The following sections discuss the different applications of plasmonic NPs for EV molecular detection and analysis based on the mechanisms of signal readout. 

### 2.1. SPR Sensing

Traditional SPR sensing is commonly performed using Au-coated SPR sensor chips [57]. Typically, the chip is directly functionalized with detection antibodies, to recognize target-specific surface protein markers on EVs [58]. Alternatively, EVs are molecularly detected in a sandwich assay, where the vesicles are first captured on the sensor chips via EV markers such as tetraspanin CD9 and CD63 for exosomes, and the targeted surface markers are then detected by the detection antibodies [59]. The functionalization of the chip surface induces a slight background response of the sensor surface. When EVs are introduced into the sensor device, a large SPR spectra shift is generated, due to the binding of the anchored detection antibodies to the targeted surface marker on the cancer EVs [58]. Due to the variance in the expression level, the binding of different surface markers induces different degrees of SPR spectra shift, which allows for specific detection of different markers or exosomes from different origins [58]. The response is fast, usually within 5 min of sample introduction. The SPR methods can detect exosomes in lower than 10^6^/mL concentrations, and their ability to analyze clinical samples for cancer diagnosis has also been demonstrated for cancer diagnosis [59]. The sensor response can also be detected by a charge-coupled device (CCD) camera, a method called SPR imaging (SPRi) [60]. Recently, Liu et al. reported a small and compact intensity-modulated SPR biosensor (25 cm × 10 cm × 25 cm) for exosomal protein detection in clinical settings [61]. This sensor consisted of a single prism, a small rotation stage, a continuous wave solid-state laser at 785 nm, and a splitter, while they used epidermal growth factor receptor (EGFR) and programmed death-ligand 1 (PD-L1) as biomarkers. Using this compact SPR sensor, the group were able to detect the exosomal EGFR derived from non-small cell lung cancer (NSCLC) at a concentration of 2 × 10^10^ exosomes/mL.

To enhance the sensitivity and throughput of SPR techniques, Lee, Weissleder, and colleagues developed a landmark nano-plasmonic exosome (nPLEX) sensor in 2014, by engineering a periodic nanohole array device [32]. This sophisticated sensor used the transmission SPR spectrum of periodic nanohole arrays and offered improved sensitivity due to the electromagnetic enhancement at the nanoholes. The chip consisted of a lattice of 44 × 32 nanoholes on a 200 nm thick Au film on a glass substrate, where each hole was 200 nm in diameter at a hole-to-hole periodicity of 450 nm. Detection antibodies were attached to the chip via a polyethene glycol (PEG) (molecular weight: 2000) linker. Exosomes were injected into the flow cell and the associated spectral shift was measured to sense target-specific proteins on the exosomes. This nPLEX sensor produced sensitivities of 10^3^ exosomes, which was 10^4^ times higher than Western blot and 10^2^ times higher than ELISA. The captured exosomes were additionally labeled with spherical AuNPs, improving the signal by 20 percent. Star shaped NPs were also used for secondary labeling, producing an enhancement of 300 percent over non-labeled exosomes. This high throughput analysis was achieved by integrating the nPLEX chip with a multi-channel microfluidic cell, where each channel could span three sensing units. The devices can measure transmission SPR intensities of 12 × 3 nanohole arrays simultaneously using the imaging setup. Using nPLEX and ascites samples from ovarian cancer patients, the authors identified exosomal CD24 and EpCAM as biomarkers for ovarian cancer diagnostics.

**Figure 2:**
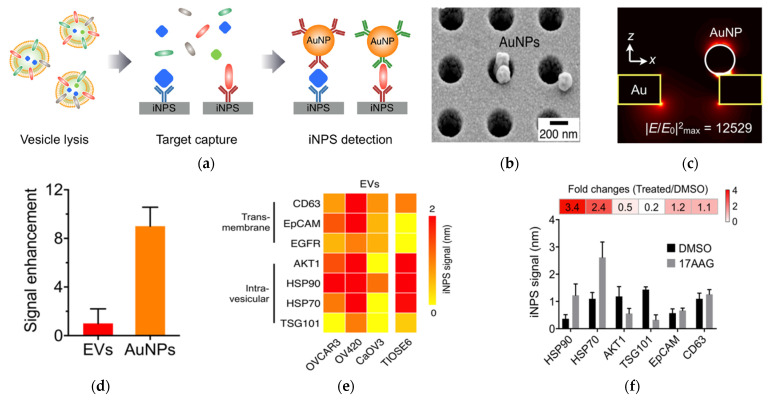
AuNP−amplified iNPS assay for the detection of intravesicular and transmembrane proteins. (**a**) Schematic of the methodology for EV protein detection. EVs are lysed to release proteins. Targeted proteins are captured on the iNPS chip via affinity ligands and detected by antibodies on AuNPs. (**b**) Scanning electron microscope image showing AuNPs on the iNPS sensor. (**c**) Finite difference time−domain (FDTD) simulation showing the concentration of the electrical fields on AuNPs. (**d**) Comparison of the signals with and without AuNPs on the iNPS sensor. (**e**) Protein profiling of EVs derived from ovarian cells. (**f**) Drug (HSP90 inhibitor) response monitoring of OV90 cells by EV protein detection with iNPS. The fold change is the protein level changes monitored by the iNPS spectral shifts after drug treatment. Reprinted with permission from [62]. Copyright (2018) American Chemical Society.

Four years later, the same researchers developed this nanohole-based SPR assay into a platform that can analyze both intravesicular and transmembrane proteins (Figure 2) [62]. This intravesicular nanoplasmonic system (iNPs) involves vesicle lysis, to release intravesicular proteins, binding of the proteins to antibody-functionalized iNPs chip, and further labeling of the captured proteins by AuNPs, to enhance the SPR shift. The AuNPs had 9-fold higher responses compared to EV binding without AuNPs. The iNPs only required 0.5 µL sample to analyze each marker and allowed for simultaneous analysis of 100 markers (10 × 10 array). Using iNPs, they were able to monitor drug responses and found unique drug-dependent EV protein signatures.

Other ways to enhance SPR sensitivity with nanotechnology have also been reported [63,64,65,66]. For example, Thakur made a self-assembly gold nano-islands (SAM-AuNIs) chip as an SPR sensor, by annealing an Au-coated glass slide [63]. This sensor achieved a LOD of 0.194 μg/mL and linear dynamic range of 0.194–100 µg/mL. It successfully detected and distinguished exosomes from microvesicles from cells, serum, and urine in a mouse model. Rather than making nanostructures on Au film, Wang et al. demonstrated an assay to enhance the traditional SPR sensor using a dual AuNP-assisted signal amplification approach, in which aptamer/T30 linked AuNPs were bound to the targeted surface proteins on the captured exosomes on the SPR chip, and then A30-coated AuNPs were applied to bind to the aptamer/T30 linked AuNPs [64]. This assay achieved a LOD of 5 × 10^3^ exosomes/mL, which was 10^4^-fold more sensitive than ELISA. An AuNP-enhancement mechanism was also achieved for the SPRi sensors [66]. Recently, multiplexed detection with an AuNP-enhanced SPRi assay was reported by Fan et al., by engineering a microarray SPRi chip [67]. This assay achieved a LOD of 10^7^ exosome/mL.

### 2.2. LSPR Light Scattering Detection

The use of AuNPs to detect exosome surface protein markers based on LSPR scattering properties was first demonstrated by Liang et al. in 2017 [33]. This nanoplasmon-enhanced scattering (nPES) assay used antibody-conjugated spherical AuNPs to target one marker, such as CD63, and antibody-conjugated AuNRs to target another marker, such as CD9. As the spherical AuNPs scatter light in the visible region and AuNRs scatter in the NIR region, they are detected as green and red particles, respectively, under dark field imaging. Thus, the two targeted surface markers on exosomes could be simultaneously detected. Moreover, when the spherical and rod-shaped AuNPs were close to each other, their LSPR exerted coupling, leading to a yellow color with increased scattering intensity. The appearance of yellow particles suggested the co-expression of the two surface markers on the same exosome. The sensitivity was very high because AuNPs can be detected at single particle level under dark field imaging. Combing a purification-free exosome capture strategy with CD81 antibodies and a multi-well glass slide as the sample sensor, this nPES assay is easy-to-use and consumes low sample volumes (1 µL of plasma). Using an nPES assay, they identified ephrin type-A receptor 2 (EphA2) as an EV marker for diagnosis of pancreatic cancer. They also demonstrated that EphA2-posive EVs can be used to monitor tumor progression and treatment response, with better performance than the conventional ELISA.

**Figure 3:**
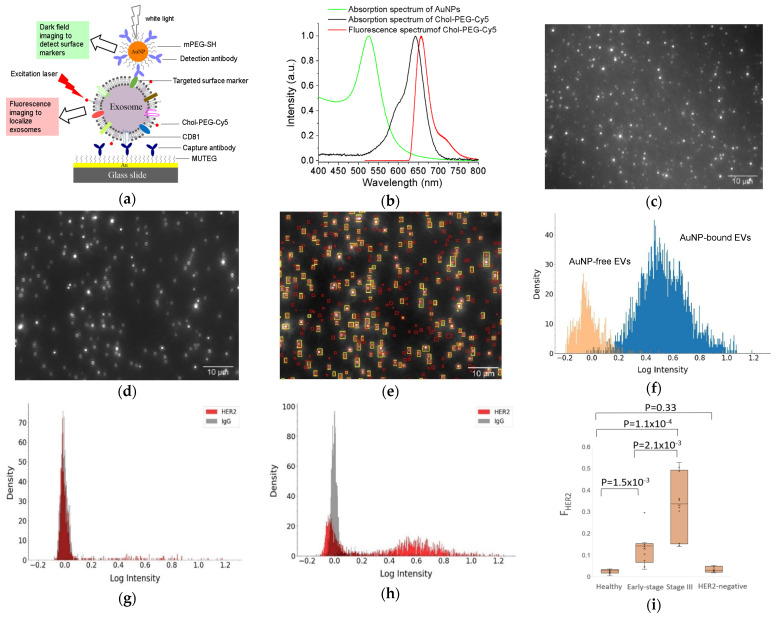
DISVT for surface protein detection of single EVs. (**a**) Schematic of the DISVT principle showing exosome capture with CD81 antibodies, membrane labeling with lipophilic Chol–PEG−Cy5, and surface protein labeling with antibody-conjugated AuNPs. (**b**) Optical properties of AuNPs and Chol–PEG−Cy5. (**c**) Fluorescence image of CD81−captured plasma EVs from a stage III HER2-positive breast cancer patient after dual labeling with Chol–PEG−Cy5 and HER2/AuNPs. (**d**) Dark field image of (**c**). (**e**) Superimposed image of (**c**) and (**d**) showing AuNP-bound EVs (overlapped red and yellow labels) and AuNP−free EVs (red labels only). (**f**) EV population density histogram showing the AuNP−bound EVs (blue peak) and AuNP−free EVs (orange peak). (**g**) EV population density histogram of EVs from a stage I HER2−positive breast cancer patient. (**h**) EV population density histogram of EVs from a stage III HER2−positive breast cancer patient. (**i**) Box plot showing the diagnostic potential of DISVT for early detection of HER2−positive breast cancer (n = 10 for each group). Reprinted with permission from [68]. Copyright (2023) American Chemical Society.

Recently, we developed a facile method to detect surface proteins on individual exosomes, using AuNPs coupled with a dual imaging approach (Figure 3) [68]. In this method, small exosome-like EVs were captured onto a multi-well Au chamber slide via CD81 antibodies and labeled with cholesterol-PEG-Cy5 (Chol-PEG-Cy5, molecular weight 2000) and localized by fluorescence imaging with an angled laser excitation. Targeted specific surface proteins on EVs were labeled with antibody-conjugated 60 nm AuNPs and detected using dark field imaging. Dual mask (EV) and target (surface protein) images were achieved with a dual fluorescence and dark field imaging system at single EV and single particle level. Superimposing the mask and target images identified AuNP-bound EVs and AuNP-free EVs. Image analysis with a python-code-based single vesicle dual imaging analysis (SEDIA) method gives the EV population density profile, which shows the AuNP-bound and AuNP-free EVs. Calibration with AuNPs linked with IgG control, the dual imaging single vesicle technology (DISVT), determines the fraction of marker-targeted EVs of all captured EVs. Using DISVT, we demonstrated that DISVT, but not ELISA, detected human epidermal growth factor receptor 2 (HER2)-positive breast cancer by quantification of HER2-positive EVs among the CD81-positive plasmon EVs. This DISVT needs less than 10 µL of 20–1000 times diluted plasma per assay. The SEDIA can analyze multiple pre-processed images simultaneously and within seconds. Due to its advantages in terms of simplicity, sensitivity, and sample consumption, this facile and ultrasensitive DISVT holds great promises for clinical cancer diagnostics and real-time monitoring. 

### 2.3. SERS Detection

Molecular detection of EVs via SERS has been reported using two different approaches: label-free and labeled assays with SERS nanotags. In the label-free detection, EVs are deposited onto SERS substrates, often a rough metal surface or Au or Ag nanopatterns on two-dimensional substrates. Molecular Raman signals from EVs such as protein, nucleic acid, and lipid are enhanced by the plasmonic substrates and detected by Raman spectroscopy [69,70,71,72,73,74]. For example, Tirinato et al. fabricated Ag nanograins on a silicon micropillar to enhance the Raman signals from exosomes deposited onto the SERS substrates [69]. They found a difference in the SERS signals from heathy and tumor cell exosomes. While exosomes from healthy cells exhibited higher intensities of peaks corresponding to lipid vibrations, exosomes from colon cancer cells showed higher signals from RNA. 

**Figure 4:**
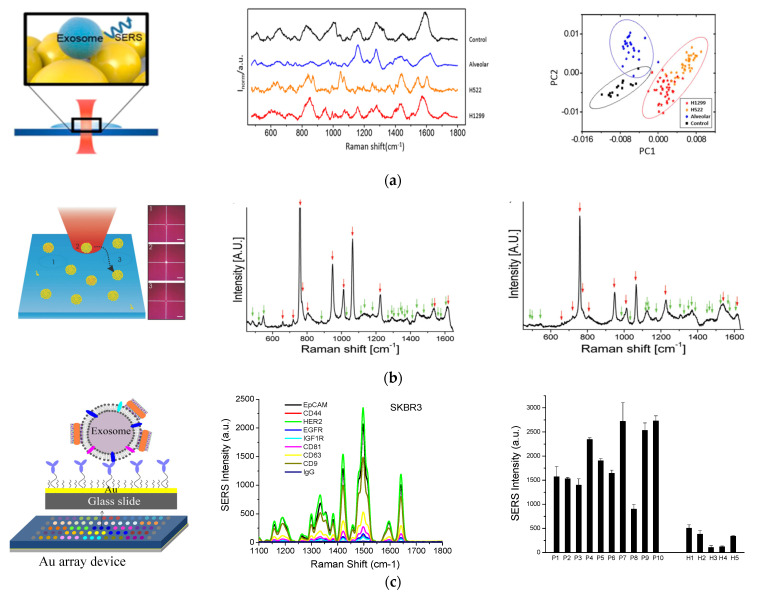
SERS—based molecular detection of exosomes. (**a**) Exosome classification through SERS detection of exosomes from normal and lung cancer cells. Left: Schematic of SERS detection of exosomes. Middle: SERS spectra of exosomes from different origins and control using PBS. Right: Principle component scatter plot showing clusters of exosomes from different origins. Reprinted with permission from [71]. Copyright (2017) American Chemical Society. (**b**) Individual molecular detection of exosome-like vesicles by label-free SERS spectroscopy. Left: Schematic of single EV detection. Each spectrum was recorded from one vesicle to another by moving the laser to a different spatial location of 1, 2, 3, etc. Middle: Representative SERS spectrum of EVs derived from B16F10 melanoma cells. Right: Representative SERS spectrum of EVs derived from red blood cells. Reprinted with permission from [75]. Copyright 2016 WILEY-VCH Verlag GmbH & Co. KGaA, Weinheim. (**c**) Molecular detection and analysis of exosomes with SERS AuNRs. Left: Schematic of the detection principle showing exosome capture with target-specific antibodies on an Au array device and exosome detection with SERS AuNRs via electrostatic interactions with exosome membranes. Middle: Detection of different surface protein markers on exosomes derived from SKBR3 breast cancer cells. Right: Detection of HER2-breast cancer by quantification of HER2-positive plasma exosomes. Cited from ref. [34].

In another study by Park et al., spherical AuNPs were deposited onto a cover glass through a drying process (Figure 4A) [71]. Purified exosomes from lung cancer cell lines and alveolar epithelial cells were deposited onto the AuNP-decorated cover glass and SERS signals from the exosomes were measured. The SERS signals from two different lung cancer cell lines, H522 and H1299, were different from each other and from the alveolar epithelial cells and the phosphate buffered solution (PBS) control. Principle component analysis (PCA) showed clear clusters of the four groups, indicating the ability to classify exosomes by SERS detection.

Label-free SERS detection on individual exosomes was achieved by Stremersch et al. in 2016 (Figure 4B) [75]. In this study, exosome-like vesicles were coated with 10 nm AuNPs utilizing electrostatic adsorption. These densely packed AuNPs enhanced the SERS signals from vesicles, which allowed for detection of individual vesicles with a Raman microscope. Using partial least squares discriminant analysis on the obtained spectra, they were able to differentiate vesicles of different origins, such as melanoma EVs and red blood cell EVs. 

Targeted surface markers on exosomes need to be detected with SERS-active nanotags via Raman reporters [76,77]. In 2018, we demonstrated a simple, inexpensive, and portable Raman exosome assay for detecting surface protein markers on exosomes using SERS AuNRs (Figure 4C) [34]. In that study, a miniaturized Au array device was fabricated for multiple analysis by assembling a 3-D printed multi-well template and gold-coated microscopy glass slide. Exosomes purified through ultracentrifugation were captured on the device using antibodies targeting surface markers of interest. To detect the captured exosomes, AuNRs were adsorbed with a QYS21 Raman tag to form SERS nanotags. The nanotags were then adsorbed onto the exosomes through electrostatic interactions between the positively charged cetyltrimethylammonium bromide (CTAB) on the surface of AuNRs and the negatively charged lipid membrane of exosomes. Using a portable Raman spectrometer, we could quantitatively detect the exosomes on the Au device and thus detect different surface protein markers using different capture antibodies. By analyzing plasma samples from healthy donors and breast cancer patients, we identified epidermal growth factor receptor 2 (HER2) and epithelial cellular adhesion molecule (EpCAM) as exosomal biomarkers for detecting HER2-positive breast cancer. 

### 2.4. Colorimetric Detection

A common point of care (POC) detection system is the lateral flow assay (LFA). The principle of LFA is based on the transportation of liquid samples via capillary force on a two-dimensional polymeric strip that molecularly interacts with the analytes in the liquid samples. The presence of the analytes is detected colorimetrically based on the absorbance properties of the detection labels and using the naked eye [78]. LFA is rapid, simple, and cost-effective, as well as suitable for the screening of many diseases [79]. A common strategy of LFA is the use of antibodies as the detection agents, which is called a lateral flow immunoassay (LFIA). In this method, the detection label, often AuNPs, is lined with antibodies to recognize the antigens to be detected [80]. As the light absorption of AuNPs is in the visible region around 520 nm, they are visible to the eye, thus making the assay widely accessible to the general public. 

The use of LFIA to detect exosomes was reported by Oliveira-Rodrı’guez et al., in 2016 [81]. In this study, anti-CD9 and anti-CD63 monoclonal antibodies were conjugated to 40 nm AuNPs to detect exosomes. Exosomes, if present in the liquid sample, were sandwiched between the AuNP conjugates and anti-CD81 immobilized in the nitrocellulose membrane of the strip, which gave a test line. The unbound AuNP conjugates migrated further, to give a control line. This method, which requires purified exosomes, can be completed within 15 min, with a LOD of 8.5 × 10^8^/mL. It has been tested for the reliable detection of exosomes in cell culture supernatants, human plasma, and urine. A year later, the same researchers achieved a multiple target assay, by incorporating multiple capture lines [82]. They also compared three different nanomaterials, AuNPs, magnetic nanoparticles (MNPs), and carbon black, for colorimetric readouts and found that the AuNPs gave the best results.

Another facile colorimetric detection platform using AuNPs is to use the color change through aggregation, as demonstrated by Jiang et al. [83]. The AuNPs were functionalized with a panel of aptamers. In the presence of exosomes, the specific binding of aptamers to the targeted surface proteins on exosomes displaced aptamers from AuNPs, inducing aggregation of AuNPs. This caused a color change from red to blue, due to the red shift of the LSPR of AuNPs from individual particles to aggregates.

A further colorimetric method is to use the enzymatic activity of AuNPs to generate colorimetric detection with 3,3′,5,5′- tetramethylbenzidine (TMB) and hydrogen peroxide (H_2_O_2_), as reported by Di et al. [84]. In this approach, AuNPs were formed on the surface of exosomes by the reduction of Au (III) chloride trihydrate (HAuCl_4_) with sodium borate hydride (NaBH_4_). In the presence of TMB and H_2_O_2_, AuNPs on the surface of exosomes catalyzed the oxidation of TMB by H_2_O_2_, inducing a color change of the solution for detection either by eye or with a absorption spectrometer. This method readily detected multiple proteins with an ELISA plate reader and differentiated hepatic cell carcinoma (HCC) patients from hepatitis B patients and healthy donors, suggesting a strong diagnostic potential. 

### 2.5. Electrochemical Detection

An electrochemical biosensor is a device that detects biomarkers by converting molecular interactions into electronic readout signals [85,86]. They have been widely used for EV detection [87,88,89,90,91,92,93]. Nanomaterials have been used in electrochemical detection systems to improve the signal readout [94,95,96]. For example, Hu et al. used AgNPs to enhance differential pulse voltammetry (DPV) signals for detection of renal binding proteins [97]. AuNPs have been widely used to enhance the electrochemical detection of DNA [98]. 

In 2016, Zhou et al. reported electrochemical detection of captured exosomes/microsomes with copper (Cu) NPs and AgNPs [99]. In their study, exosomes or microsomes were captured onto an aptamer-modified microfabricated chip containing 11 individual circular Au electrodes. Antibody-conjugated AgNPs were used to report EpCAM, and antibody-conjugated CuNPs were used to report prostate-specific membrane antigen (PSMA), a biomarker for prostate cancer. Electro-oxidation of these metal NPs induced an electronic response for signal readout. This sensor reached a LOD of 50 exosomes/sensor. Using this sensor, the authors reported that prostate cancer patients showed significantly higher levels of EpCAM and PSMA-positive exosomes, suggesting a good potential for diagnosis of prostate cancer. 

### 2.6. Other Detection Methods

Other detection methods involving plasmonic nanomaterials have also been reported [100,101,102,103,104,105,106,107,108,109,110]. One of these methods is to use cantilever arrays to respond to the molecular binding of multiple targets [100]. AuNPs coated with antibodies were used to detect exosomes captured on a microcantilever. With a reference cantilever, this method detected CD24, CD63, and EGFR on exosomes simultaneously, with a LOD of ~0.1 pg/mL.

Another method is to use AuNPs to amplify a surface acoustic wave (SAW) for the detection and quantification of exosomes, as reported by Wang et al. [106]. Exosomes were captured onto a SAW chip that was functionalized with anti-CD63 antibodies. Streptavidin-conjugated AuNPs recognized exosomes via biotin-conjugated EpCAM that was bound to the exosomes in advance. This sensor reached a high sensitivity, with a LOD of 1.1 × 10^3^ exosomes/mL. The AuNPs enhanced the signals of the SAW chip by two orders of magnitude.

A further exciting application of AuNPs for the in vivo imaging and tracking of exosomes was demonstrated by Betzer et al. in 2017 [101]. In their studies, exosomes derived from mesenchymal stem cells (MSCs) were labeled with AuNPs via active uptake of glucose-modified AuNPs. The AuNP-labeled MSC exosomes were administrated in vivo in a mouse model via intranasal (IN) or intravenous (IV) administration. Quantification of the Au amount with inductively coupled plasma (ICP) spectrometry at 24 h post-injection showed the accumulation of exosomes in a substantial amount in the brain. In an animal model of stroke, CT imaging clearly identified and tracked in real time the exosome migration and accumulation in the stroke-effected area of the mouse brain. They were detected as early as 1 h post-IN injection. This exosome labeling and in vivo imaging technique can serve as a powerful tool for the diagnosis of various brain disorders.

In addition, AuNPs can be used to work with fluorophores to detect EVs, by making use of the energy transfer process between plasmonic NPs and fluorophores [102,103]. This energy transfer leads to a quenching effect on the emission of fluorophores when the fluorophores are in proximity with AuNPs. The emission of the fluorophore is resumed when the fluorophore leaves the metal NPs. AuNPs themselves also exhibit fluorescence and thus can serve as fluorescent labels for EV detection [105]. Moreover, AuNPs can also be used as a pure scaffold to carry reporter molecules or targeting ligands for EV detection via other detection mechanisms [104].

## 3. Fluorescence Nanomaterials

### 3.1. Quantum Dots (QDs)

Quantum dots (QDs) can be used in many ways to detect EVs. One of their greatest advantages is that they are very small, being approximately 2–10 nm in diameter. This gives them the ability to be used as an efficient labeling and detection tool for small exosomes. Another advantage to using QDs is that they have strong and narrow fluorescence properties with a broad absorption spectrum. This allows for bright multicolor fluorescence imaging with a single laser excitation. In addition, they exhibit excellent photostability [111]. Furthermore, QDs functionalized with a variety of ligands are readily available from commercial resources [112].

**Figure 5:**
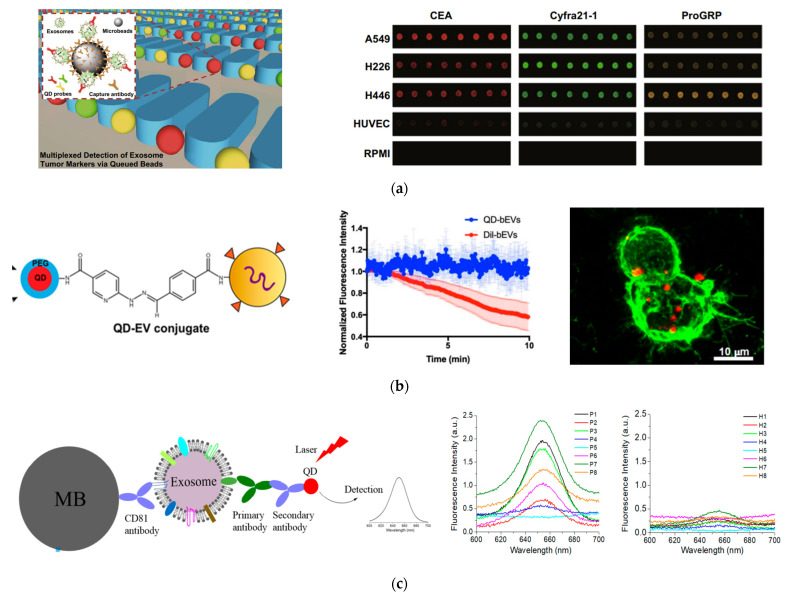
Fluorescent detection of exosomes using QDs. (**a**) Isolation and multiplexed detection of exosomal tumor markers using QDs and magnetic beads in a microarray chip. Left: Schematic of the methodology showing exosome isolation with single magnetic bead trapped in the microfluidic pillars and multiplexed detection with multicolor QDs. Right: Fluorescence images of exosomes derived from different cell lines using three different target-specific QDs: QD525, QD585, and QD625. Cited from ref. [115]. (**b**) EV labeling and visualization with QDs. Left: Schematic of EV labeling with QDs. Middle: Photostability of QDs in comparison to Dil dye. Right: Fluorescence imaging showing the distribution of QD-labeled EVs (red) on lection stained BV-2 cells (green). Reprinted with permission from [117]. Copyright (2020) American Chemical Society. (**c**) Detection of breast cancer with QDs combined with immunomagnetic separation. Left: Schematic of the immunomagnetic isolation with magnetic beads and fluorescence detection of surface protein markers by QD655. Right: Fluorescence spectrum of exosomes from HER2-positive breast cancer patients and healthy donors after labeling with HER2-targeted QDs showing diagnosis potential of QDs for detection of HER2-positive breast cancer. Cited from ref. [119].

Thus, QDs are often used as excellent fluorescent labels to detect exosomes through fluorescence imaging and spectroscopy [113,114,115,116,117,118,119,120]. One remarkable study was performed by Bai et al., who demonstrated multiplexed detection of tumor exosomes via queued beads combined with QDs in a microarray (Figure 5A) [115]. Exosomes were isolated and bound to magnetic microbeads, which were then trapped among micropillars in a microfluidic chip. Target-specific QDs were bound to exosomes on the microbead and detected by fluorescence imaging. Due to the separation of microbeads by the micropillars, fluorescence signals from QDs were dispersed into every single bead, thus avoiding optical interferences, for more accurate test results. By using QDs of different sizes and thus different colors (red, green, and yellow) with different cancer markers, they could detect exosomes derived from different cancer cell lines. Using this technique, they found that the cancer markers on exosomes derived from lung cancer cells were about six-fold higher than those from normal cell lines. Additionally, they were able to detect lung cancer with good diagnostic power using clinical plasma samples. 

A different study using QDs imaged exosomes on live cells and tissue sheets (Figure 5B) [117]. In this study by Zhang et al., QDs were attached to exosomes via click chemistry. Using this labeling technique and fluorescence imaging, they were able to visualize semen EVs from human vaginal mucosa. They were also able to visualize brain EVs in real time and observe the interactions of brain EVs with microglial BV-2 cells. This method enabled the study of EV localization and function in cells and tissue sections with bright and photostable QDs.

In our recent study, we demonstrated a simple way to detect exosome surface protein markers and to diagnose cancer, using QDs combined with magnetic separation (Figure 5C) [119]. We used magnetic beads linked with CD81 antibodies to isolate exosomes from a conditioned cell culture medium and plasma samples from human subjects. QD655 was used to indirectly target the surface caner markers of interest with secondary antibodies via streptavidin and biotin chemistry. A routine fluorimeter was used to collect the fluorescence spectrum from the exosome solutions labeled with QD655 or the control without primary antibodies. This method exhibited a good sensitivity, with a LOD of 4.7 × 10^7^ exosomes/mL. It was well correlated with ELISA, with a high Pearson correlation coefficient of 0.986. Using this method, we quantitatively determined the expression level of EpCAM, HER2, CD44, and CD24 on three breast cancer cell lines, MDA-MB-231, SKBR3, and MCF7, which were different from each other and different from that of the healthy breast cell line MCF12A. In a proof-of-concept study, with pilot clinical samples from HER2-positive breast cancer patients and healthy donors (n = 8 for each group), we showed that HER2-positive exosomes in plasma were a strong diagnostic marker for diagnosis of HER2-poisitve breast cancer, with an area under curve (AUC) around 0.97. The advantage of this method over other detection techniques is its simplicity, which only involves magnetic isolation, mixing with primary antibodies, secondary antibody-conjugated QD655, and the detection of the exosome suspension with a common fluorometer.

A different application of QDs for exosome detection was reported by Boriachek et al. in 2017 based on electrochemical detection [121]. CdSe QDs were functionalized with anti-HER2 and anti-FAM134B antibodies to target breast and colon cancer exosomes, respectively. The QDs were dissolved in acid conditions and the Cd^2+^ were quantified by anodic stripping voltammetry at a bare glassy carbon working electrode. This method was very sensitive, with a LOD of 105 exosomes/mL. QDs have also been used to enhance electrochemiluminescence signals (ECL) by self-assembly to Ru(dcbpy)_3_^2+^, to reduce energy loss and shorten the electron transfer distance [122]. MoS_2_ QDs were used to fabricate a self-powered photoelectrochemical biosensor for exosomal RNA detection, by combining with TiO_2_ nanosilks [123].

### 3.2. Carbon-Based Nanoparticles

Other fluorescence NPs have also been used to detect EVs [124,125,126,127,128]. Particularly, upconversion nanoparticles (UCNPs) are attractive for EV detection, due to their strong fluorescence properties. An example is the aptasensor reported by Chen et al. in 2018 [125]. In this sensing platform, the UCNPs worked with plasmonic AuNRs for exosome detection through luminescence resonance energy transfer (LRET) from UCNPs to AuNPs. This was done by linking the two nanoplatforms using aptamers via specific binding to the targeted molecules on the surface of exosomes. The presence of exosomes quenched the fluorescence of UCNPs, which enabled the quantitative detection of exosomes. This LRET-based mechanism achieved a LOD of 1.1 × 10^6^ exosomes/mL. The same group later replaced AuNPs with a rhodamine dye and achieved a much higher detection sensitivity, with a LOD of 8 × 10^4^ exosomes/mL [127].

A remarkable recent study by Huang et al. extended UCNPs to detect single EVs [129]. The authors found that while the Er^3+^-doped UCNPs had a better brightness, the Tm^3+^-doped UCNPs had a better resolution beyond the diffraction limit. Using a super-resolution microscope, they could distinguish adjacent UCNPs on single small EVs. They also found that the number of bound UCNPs ranged from 3 to 21 when using super-resolution fluorescence imaging. This method opens an exciting opportunity to measure the number of surface antigens on single EVs using fluorescence NPs. 

## 4. Magnetic Nanostructures

Magnetic nanostructures such as MNPs and magnetic nanowires are often used to isolate and purify EVs from cell culture supernatant and body fluids [130,131,132,133,134,135,136,137,138,139,140,141,142,143]. However, they can also be used as signal readout agents to the detect molecular constitutes of EVs. A major approach was developed by Lee, Weissleder, and co-authors, who also developed the nanoplasmonic sensors described above [144]. They designed a microfluidic chip that can isolate and concentrate MNP-bound EVs and also provide in-line detection, via a micro-nuclear magnetic resonance (µNMR) device that they developed previously for cancer cell detection [145,146,147]. The principle of µNMR is based on the measurement of the transverse decay rate (R2), which is proportional to the MNP concentration. This allows quantification of the targets of interest via MNP-labeling. To detect microvesicles with µNMR, they labeled the vesicles with target-specific MNPs via a two-step biorthogonal approach. To calibrate the vesicle total counts, they used CD63 as an internal reference marker. The signal linearly corresponded with the amount of microvesicles. The µNMR method was highly sensitive, being 10^4^, 10^3^, and 10^2^-fold more sensitive than Western blotting, ELISA, and NTA, respectively. Using µNMR with MNPs as the detection agents, they confirmed that the protein profile of microvesicles from glioblastoma multiforme (GBM) indeed reflects that of their parental cells. Using the µNMR system, the authors found that circulating GBM microvesicles can serve as a surrogate for tumor mutations and monitor treatment-induced changes.

**Figure 6:**
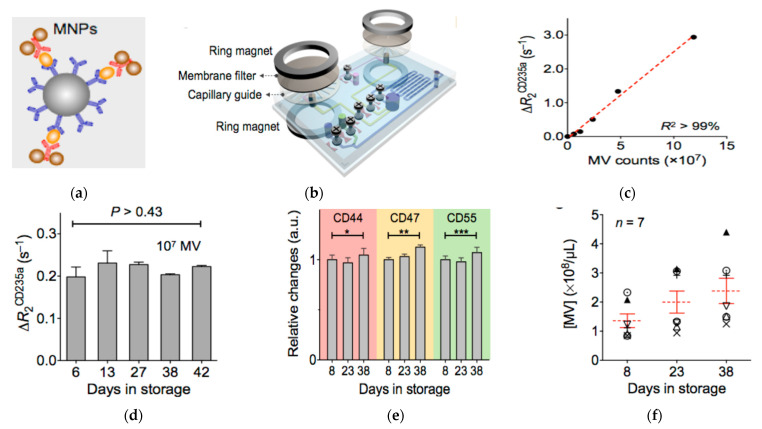
Magnetic nanosensor for the detection and profiling of microvesicles derived from red blood cells (RBC). (**a**) Schematic of microvesicle labeling with MNPs. (**b**) Schematic of the µNMR system showing the derived sensor coupled with membrane filter for microvesicle separation. (**c**) Relaxation rate change (ΔR_2_CD235a) versus the number of microvesicles using CD235a targeted MNPs. (**d**) ΔR_2_CD235a at different days of red blood cell storage showing stable vesicle expression of CD235a. (**e**) Relative changes in the expression levels of CD44, CD47, and CD55 during storage of red blood cells showing little changes in the expression levels of these proteins on microvesicles. * *p* > 0.48; ** *p* > 0.15; *** *p* > 0.17. (**f**) Microvesicle concentration over storage time showing the increase of the amount of microvesicles with storage time. Reprinted with permission from [148]. Copyright (2013) American Chemical Society.

Lee, Weissleder, and co-authors later developed another μNMR system integrated with online isolation (Figure 6) [148]. The μNMR system had a membrane filter and capillary guide sandwiched between two ring magnets, to isolate microvesicles from blood. After isolation, the microvesicles were collected in a reservoir and labeled with MNPs, which were then detected by measuring the transverse relaxation rate. The whole process was performed entirely on the chip, eliminating the need for external vesicle operations. Using this μNMR system, the authors found that the counts of microvesicles increased with the aging of blood, but the levels of protein expression, such as for CD235a, CD44, CD47, and CD55, were the same. They also found that microvesicles had the ability to induce oxidative stress and consumed nitric oxide. This suggests that this system can be used to interrogate and monitor blood quality and blood transfusion safety.

Similarly to other nanomaterials, MNPs can also be used as a nanocarrier of bioactive molecules for exosome detection. For example, Chen et al. functionalized Fe_3_O_4_ NPs with EpCAM-specific aptamers [149]. In the presence of exosomes, the aptamers on the Fe_3_O_4_ NPs bound to the exosomes and thus were displaced from the NPs. The aptamer-bound Fe_3_O_4_ NPs had a higher peroxidase activity than untreated ones. Thus, the presence of exosomes decreased the catalytic activity of Fe_3_O_4_ NPs, which led to a lower degree of oxidation of TMB by H_2_O_2_ than the control without exosomes or with exosomes bearing a low level of EpCAM. This colorimetric change can be used to quantitatively measure the level of targeted exosomes. MNPs can also be used for direct colorimetric detection of EVs via LFIA using the brown color of the NPs [150].

## 5. Organic Frameworks

### 5.1. Metal Organic Frameworks (MOFs)

Metal-organic Frameworks (MOFs) are a category of 2-D and 3-D synthesized organic and crystalline nanomaterials formed from the self-assembly of organic linkers and metal ions [151,152,153]. MOFs are highly tunable, porous, rigid, and stable materials. In some cases, MOFs can expand and contract based on their environment and the material loaded within the pores [151,152,153]. MOFs are used in various applications, such as solar panels, carbon capture, drug delivery, and cancer detection [154].

**Figure 7:**
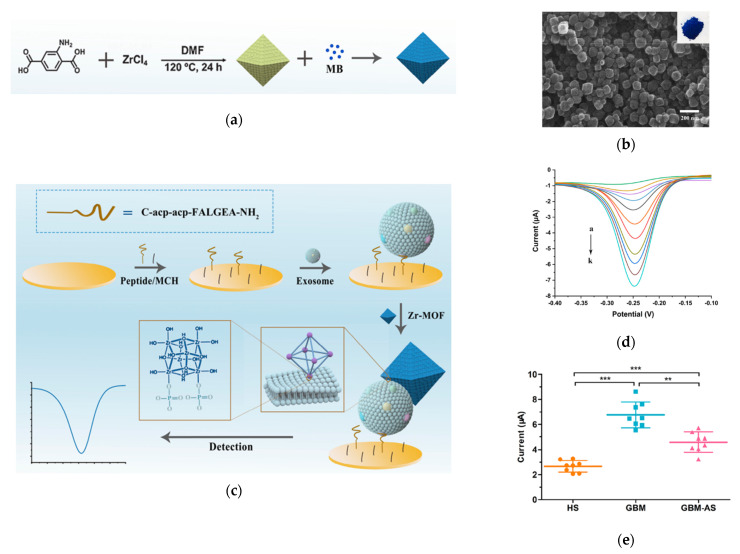
Detection of Glioblastoma-derived exosomes with Zr−based MOFs. (**a**) Schematic of the preparation of MB@UiO−66 nanoprobes. (**b**) Scanning microscope image of MB@UiO−66 nanoprobes. (**c**) Schematic of the principle of electrochemical detection of exosomes using MB@UiO−66 nanoprobes. (**d**) SWV recorded with different concentrations of exosomes. (**e**) Scattered dot plots of exosomes from different human subjects. HS: healthy subjects. GBM: Glioblastoma. GBM−AS: GMB after surgery. ***: *p* < 0.0001. **: *p* < 0.001. Error bars represent the standard deviation of three independent experiments. Reprinted with permission from [155]. Copyright (2020) American Chemical Society.

A novel application of MOFs is to build an electrochemical biosensor for exosome detection, as demonstrated by Sun et al. in 2020, by making use of the high porosity property of MOFs (Figure 7) [155]. In this technique, highly porose UiO-66-NH_2_, a typical Zr-MOF, was synthesized and loaded with electroactive redox indicator methylene blue (MB), which is often used for electrochemical detection [156]. A specially-designed peptide was assembled on the electrode surface to recognize EGFR and EGFR variant (v) III mutation (EGFRvIII), the GBM protein biomarkers, so as to capture GBM exosomes. Then, the MB@Zr-MOFs bound to the captured exosomes, producing a highly amplified electrochemical signal via the interaction between the Zr^4+^ from the MOFs and phosphate head of the lipid membrane from the exosomes. This electrochemical signal, recorded by the square wave voltammograms (SWV) was linearly proportional to the concentration of the bound exosomes. This sensor exhibited a LOD of 7.83 × 10^6^ exosomes/mL. Using this sensor, the authors distinguished GBM patients from healthy groups by detecting and quantifying EGFR/EGFRvIII positive exosomes from serum samples.

A portable paper-based electrochemical biosensor using Zr-MOFs was later reported by Liu et al. [157]. This sensor was developed by combing a Zr-MOF functionalized paper and a screen-printed electrode (SPE). Zr-MOFs were coated on the surface of the paper sensor to capture exosomes via the Zr-P-O interactions. Then, an aptamer was added to recognize CD63. The aptamer also triggered hybridization chain reaction (HCR) amplification, which then catalyzed the oxidation of TMB and thus reduced the signal response of TMB on SPE. Due to the HCR, this sensor is highly sensitive, with a LOD of 5 × 10^3^ exosomes/mL. A different DNA amplification strategy involving MOF was reported by Cao et al. [158]. They made a pH-sensitive (via polyvinylpyrrolidone (PVP)) and horseradish peroxidase (HRP)-encapsulating zeolitic imidazolate framework-8 (ZIF-8). Hyperbranched rolling circle amplification (HRCA) was introduced on the capture probe of exosomes to release protons and thus lower the pH of the local environment where the PVP@HRP@ZIF-8 was located. At low pH, the MOF complex released HRP, to amplify electrochemical signals. This sensor reached a superior sensitivity, with a LOD of 334 exosomes/mL. 

By varying the strategies in the signal readout and the MOF function, different electrochemical sensors can be developed for EV detection, in terms of both protein and RNA markers [159,160]. By combining other detection approaches, MOFs can be used to capture and purify exosomes with or without MNPs [141,161,162,163]. For example, an MOF made of Tim4@ILI-01 immunoaffinity flake, has the ability to capture and separate exosomes in samples from healthy patients and those diagnosed with lung adenocarcinoma [161]. The Fe_3_O_4_@UiO-66-NH_2_@PA-Ti^4+^ exhibited an exosome capture and recovery rate of 97.3% within 5 min of exosomes [141]. 

### 5.2. Carbon Organic Frameworks (COFs)

Covalent organic frameworks (COFs) have started to garner interest for EV detection and capture in recent years, due to their high porosity. COFs are 2-D and 3-D crystalline structures exhibiting strong covalent bonds producing a tunable, porous, and extremely stable material. Unlike MOFs, COFs do not contain metal but are constructed through covalently bonded organic materials [164]. A recent work by Wang et al. demonstrated the use of COFs for the detection of colorectal cancer exosomes [165]. In this work, a COF-based nanoprobe was fabricated by complexing para-sulfocalix arene hydrate (pSC_4_), AuNPs and HRP. This HRP-pSC_4_-AuNPs@COFs was bound with amino acids on the exosome surface via pSC_4_. The high porosity of COFs ensured the high loading amount of HRP. The AuNPs boost the migration of charge carriers and the response of biosensors for exosome detection. Due to these unique features, this nanoprobe exhibited a high LOD of 1.6 × 10^5^ exosomes/mL. It successfully differentiated exosomes between colorectal cancer patients and heathy controls.

## 6. Carbon Nanomaterials

### 6.1. Carbon Nanotubes (CNTs)

Carbon nanotubes (CNTs) are nanoscale tubes made of carbon bonds [166]. The two categories of carbon nanotubes are single-wall carbon nanotubes (SWCNTs) and multi-wall carbon nanotubes (MWCNTs) [166,167]. CNTs have been used for many applications, from catalyst supports, to biochemical sensors and drug delivery carriers [168,169,170,171]. By making CNT arrays, exosomes can be isolated and purified for downstream applications [172]. 

**Figure 8:**
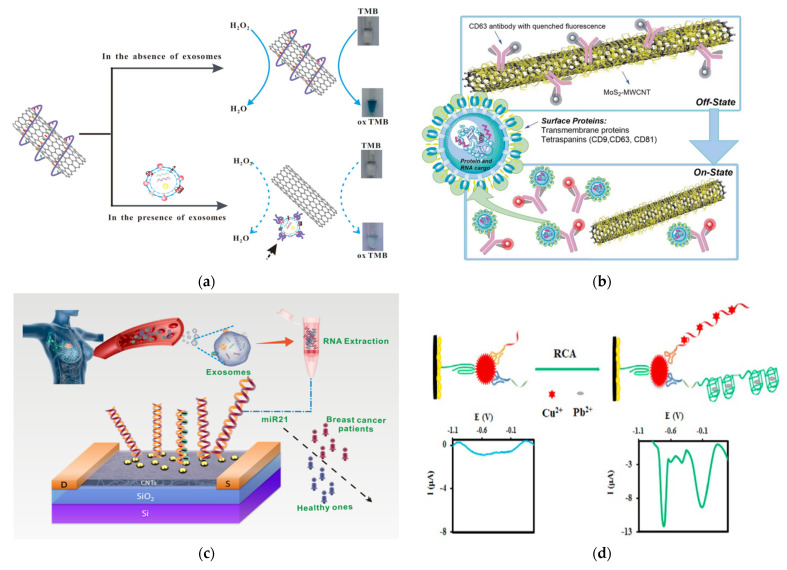
Exosome detection with CNTs. (**a**) Schematic of a colorimetric aptasensor based on DNA−capped SWCNTs for exosome detection. Reprinted with permission from ref. [173]. Copyright@ 2017 Elsevier B.V. (**b**) Schematic of the detection mechanism of a MoS_2_−MWCNT-based nanosensor for exosome detection and quantification. Reproduced from ref. [174] with permission from the Royal Society of Chemistry. (**c**) Schematic of the detection of exosomal miRNA21 using DNA-functionalized CNT FET biosensor. Reprinted with permission from [175]. Copyright {2021} American Chemical Society. (**d**) Schematic of an electrochemical aptasensor for the multiplexed detection of exosome biomarkers. Reprinted with permission from ref. [178]. Copyright@ 2017 Elsevier.

An interesting application for EV detection was demonstrated by Xia et al., who developed a colorimetric sensor by confirming the surface chemistry of SWCNTs (Figure 8A) [173]. In this method, a CD63-specific aptamer was conjugated to SWCNTs, to improve the peroxidase activity of SWCNTs. This induced a bright blue color from the TMB solution. When the SWCNTs were exposed to exosomes, the aptamers bound to CD63 on exosomes and are thus were disposed from the SWCNTs. This resulted in a reduction in the peroxidase activity of the SWCNTs and thus a reduction in the color intensity of TMB. Although this method is not super sensitive (LOD = 5.2 × 10^8^ exosomes/mL), it is very simple and easily accessible to the general public, as it can be detected using the naked eye or regular a UV-Vis spectrometer.

Another interesting application is to use CNTs as a quencher to develop a fluorometric nanosensor for exosome detection and quantification, as shown by Tayebi et al. (Figure 8B) [174]. In this method, MoS_2_-MWCNT was modified with PE-conjugated CD63 antibody. The fluorescence of PE on the CD63 antibody was quenched using the MoS_2_-MWCNT in the absence of exosomes. In the presence of exosomes, the PE-conjugated CD63 antibody binds to exosomes and thus leaves the MoS_2_-MWCNT, which recovers the fluorescence of PE molecules, so as to quantitatively measure the level of exosomes with targeted surface protein markers.

Li et al. have developed a field-effect transistor (FET) biosensor for the detection of miRNA from tumor exosomes by making a polymer-sorted high purity semiconducting CNT film on a Si/SiO_2_ substrate [175] (Figure 8C). On top of the CNT film, a thin film (3 nm) of yttrium was deposited by electron beam evaporation (EBE) and oxidized to yttrium oxide, to form an insulating layer. Then, Au was deposited by EBE to form AuNPs to anchor the oligonucleotide. This oligonucleotide probe binds to miRNA via hybridization, to induce current changes, allowing the detection and quantification of exosomes. This FET sensor achieved a sensitivity of 0.87 aM miRNA.

CNTs can also be used to develop electrochemical-based EV sensors. For example, Si et al. reported electrochemcial detection of lung cancer exosomes using DNA/ferrocene-modified SWCNT [176]. Sahraei et al. developed a biodegradable electrochemical paper-based device (ePAD) by incorporating synthetic carbon, a silver ink screen, and AuNPs on paper [177]. CD9 antibodies were coordinated on the surface of the nanocomposites for detection. This sensor exhibited superior sensitivity, with a LOD of 7 × 10^4^ exosomes/mL. In the studies by Hashkavayi, MWCNT, ionic liquid, and chitosan were incorporated onto a screen-printed carbon electrode and modified with CD63, to capture exosomes (Figure 7D) [178]. Then, the EpCAM or HER2 positive exosomes bound to a EpCAM or HER2 aptamer, with primer sequences that acted as a rolling circle amplification (RCA) reaction initiator. After RCA, the addition of copper and lead ions led to strong electrochemical signals that were measured by DPV due to their complexation with the RCA products. 

### 6.2. Carbon Nanosheet

Graphitic carbon nitride nanosheets (g-C_3_N_4_ NSs) are two-dimension nanomaterials with graphene-like properties, which have been used for various applications, from materials science to disease diagnostics [179,180,181]. An example using g-C_3_N_4_ NSs for EV detection was demonstrated by Wang et al., who exploited the peroxidase-like activities of the nanosheet for colorimetric detection of exosomes [182]. Without exosomes, the single stranded DNA (ssDNA) adsorbed on g-C_3_N_4_ NSs enhanced the catalytic activity of the nanosheet, and thus TMB was strongly oxidized by H_2_O_2_, to give an intense blue color. In the presence of exosomes, however, the ssDNA binds to the targeted surface proteins on exosomes. Thus, the catalytic activity of the g-C_3_N_4_ NSs was not enhanced and the TMB oxidation by H_2_O_2_ was only moderate. Under this situation, the color of the oxidized TMB was weaker than the control without exosomes. This sensor was sensitive, capable of differentiating the level of CD63 expression on exosomes between MCF7 breast cancer cells and MCF-1-A normal cells. 

### 6.3. Graphene

Graphene is a two-dimension sheet of SP^2^ hybridized carbon atoms [183]. The long range pi conjugation in graphene leads to outstanding electrical, thermal, and mechanical properties [184]. These properties have been recently used for molecular detection and analysis of circulating biomarkers including EVs [185]. The electrical property of graphene and graphene oxide provide an excellent opportunity to design field-effect-transistor (FET)-based biosensors for EV detection [186,187,188,189,190]. For example, Tsang et al. fabricated a back-gated graphene FET biosensor by depositing a graphene monolayer onto a microfluidic device by chemical vapor deposition [186]. Functionalization of the graphene surface with CD63 antibodies captured the exosomes. The microfluidic channel separates the graphene into sample touched and sample-free areas. These two areas exhibit different electric properties and can sensor the binding of exosomes in the solution using the current. This sensor exhibited a LOD down to 0.1 µg/mL. In another study, Yu et al. fabricated a reduced graphene oxide (RGO) FET, functionalized with CD63 to capture and detect exosomes [187]. This method achieved a low LOD of 3.3 × 10^4^ exosomes/mL. This label-free technique can directly quantify exosomes in serum samples and has shown diagnostic potential for prostate cancer. Additionally, the porous structure has been used to isolate exosomes with and without other isolation materials and devices [191,192,193]. 

## 7. Other Nanomaterials

In addition to the nanomaterials discussed above, various other nanomaterials have also been reported including polymer nanowires [194], DNA nanomaterials [195,196,197,198,199,200,201], fluorescent metal compound NPs [202], and lipid-based NPs [203]. For example, He et al. developed a aptamer-based fluorescence DNA nanodevice (ABDNs) to visualize and quantify exosomes in plasma samples at single vesicle level [201]. The high sensitivity was driven by the HCR of the aptamer probe on the surface of exosomes using fluorescence molecular hairpins. The HCR led to nanoscale DNA long chains on the surface of the exosomes, allowing fluorescent detection of single exosomes. In another DNA-based platform by Zhao et al., exosomes with aptamers were detected through the detection of exosomal DNAs [199]. Target-specific recognition was amplified by the movement of a 3-D DNA walker and then detected by an electrochemical ratiometric sensor. This approach achieved a LOD of 1.3 × 10^4^ exosomes/mL.

## 8. Summary and Perspective

We have summarized and discussed recent analytical technologies and devices that made use of classic nanomaterials for the detection of proteins and nucleic acids of exosomes and microvesicles (Table 1). These nanomaterials were used either alone or with other nanomaterials or molecules for molecular EV detection, with different mechanisms such as light scattering imaging, fluorescence detection, and electrochemical sensing. The detection sensitivity had a varied ranged, from 10^8^ EVs/mL down to single EV, depending on the type of nanomaterials and the signal readout. The most widely used nanomaterials were plasmonic NPs, due to their wide range of properties, including plasmonic sensing, light scattering, SERS, and fluorescence.

While nanomaterials and associated technologies are promising and have significant advantages over other existing technologies, they also have some limitations. One generic limitation is the challenge in the detection of small EVs at single vesicle level. Except QDs, the size of NPs is usually large and more than 20 nm. Attachment with target ligands such as antibodies further increases the particle’s size. However, EVs can be as small as 30 nm. In our studies, we showed that only one AuNP of 60 nm in diameter can bind to an EV of 40 nm in diameter [68]. They cannot detect EVs smaller than 40 nm. Such sized AuNPs cannot be used to quantify a number of the targeted surface proteins. The additional limitations of each type of nanomaterial-based technique depend on the structural and functional properties of nanomaterials. For example, the µMR devices using MNPs are sophisticated and require specialized skills and personnel to operate; similarly to the electrochemical sensors that require expertise in electric chemistry. The colorimetric detection approaches are easy and inexpensive, but they typically have a limited sensitivity.

Despite these limitations, the popularity of nanomaterials for molecular EV detection and analysis is expected to rise rapidly, owing to the unique structural and functional properties of nanoscale materials and devices. In particular, research is going to focus on single EV detection. This is because EVs are heterogeneous, and disease-derived EVs are mixed with a vast background of non-tumor EVs from various tissues and hematopoietic cells. The bulk methods are inherently contaminated with normal exosomes, which masks EV heterogeneity and limits the sensitivity of detection in analyzing clinical samples. To more sensitively, precisely, and quantitatively detect, monitor, and investigate cancer EVs, EVs need to be analyzed at the single vesicle level. Different single vesicle technologies have been reported in recent years [204]. We expect nanomaterials will play a rapidly increasing role in the development of new SVTs for EV protein and nucleic acid detection and profiling, for basic vesicle research and clinical applications.

## Figures and Tables

**Table 1:** Summary of the major nanomaterials for molecular detection and analysis of extracellular vesicles.

Type of Nanomaterials	Material Composition	Detection Mechanism	Lowest LOD	Clinical Test	Reference
Plasmonic NPs and nanopatterns	AuNPs, nanostar, nanoisland, nanowhole array	SPR	3000 exosomes	Ovarian, prostate, and lung cancers	[32,62,63,64,65,66,67]
	AuNPs, AuNRs	Light scattering	Single vesicle	Pancreatic and breast cancers	[33,68]
	AuNPs, AuNRs, Ag nanograins, Ag nanocube, Au-Ag core-shell NPs	SERS	Single vesicle	Breast and pancreatic cancers	[34,69,70,71,72,73,74,75,76,77]
	AuNPs	Colorimetric	10^8^ exosomes/mL	Liver cancer	[81,82,83,84]
	AgNPs, CuNPs	Electrochemical	50 exosomes/sensor	Prostate cancer	[99]
	AuNPs	Acoustic, mechanic, CT, fluorescence	1000 exosomes/mL	Breast and lung cancers	[100,101,102,103,104,105,106,107,108,109,110]
Fluorescence NPs	QDs	Fluorescence	10^5^ exosomes/mL	Breast, colorectal, lung, and pancreatic cancers	[113,114,115,116,117,118,119,120]
		Electrochemical	10^5^ exosomes/mL	N/A	[121,122,123]
	UCNPs and other carbon-base NPs	Fluorescence	Single vesicle	N/A	[124,125,126,127,128,129]
Magnetic NPs	IO NPs	µNMR	10^4^ microvesicles	Glioblastoma	[144,148]
	IO NPs	Colorimetric and others	3.6 × 10^9^ exosomes/mL	Prostate cancer	[149,150]
Organic framework NPs	MOFs	Electrochemical	334 exosomes/mL	Glioblastoma, breast, and lung cancers	[155,156,157,158,159,160]
	COFs	Colorimetric	1.6 × 10^5^ exosomes/mL	Colorectal cancer	[165]
Carbon nanomaterials	CNTs	Colorimetric	5.2 × 108 exosomes/mL	Breast	[173]
	CNTs	Fluorescence	1.5 × 10^6^ exosomes/mL	N/A	[174]
	CNTs	FET	0.87 aM exosomes	Breast cancer	[175]
	CNTs	Electrochemical	1 exosome/mL	N/A	[176,177,178]
	g-C_3_N_4_ NSs	Colorimetric	1.4 × 10^9^ exosomes/mL	Breast cancer	[182]
	Graphene	FET	9 × 10^3^ exosomes/mL	Prostate and breast cancers	[186,187,188,189,190]
Other nanomaterials	Conductive polymer NWs	Electric	N/A	Lung cancer	[194]
	DNA NPs	Fluorescence	N/A	N/A	[195]
	DNA NPs	Electrochemical	9.5 × 10^2^ exosomes/mL	Breast cancer and leukemia	[196,197,198,199,200,201]
	CuS NPs	Chemiluminescence	10^4^ exosomes/mL	Breast cancer	[202]
	Lipid-based NPs	Fluorescence	N/A	Lung cancer	[203]

## Data Availability

Not applicable.
